# Withdrawal: Shaping environmental responsibility through virtual wilderness: The key role of aesthetic psychology

**DOI:** 10.1371/journal.pone.0335284

**Published:** 2025-10-13

**Authors:** The PLOS One Editors

*PLOS One* has withdrawn this article [1] because it was published in error. The article contents were removed from the journal website on October 9, 2025. The publisher apologizes for the error.
